# A simple procedure for the comparison of covariance matrices

**DOI:** 10.1186/1471-2148-12-222

**Published:** 2012-11-21

**Authors:** Carlos Garcia

**Affiliations:** 1 Department Xenética, CIBUS Campus Sur, Universidade de Santiago de Compostela, Santiago de Compostela, Galicia 15782, Spain

**Keywords:** Eigenvectors, Principal component analysis, Littorina Saxatilis, Matrix orientation, Matrix shape, Hybrid zone

## Abstract

**Background:**

Comparing the covariation patterns of populations or species is a basic step in the evolutionary analysis of quantitative traits. Here I propose a new, simple method to make this comparison in two population samples that is based on comparing the variance explained in each sample by the eigenvectors of its own covariance matrix with that explained by the covariance matrix eigenvectors of the other sample. The rationale of this procedure is that the matrix eigenvectors of two similar samples would explain similar amounts of variance in the two samples. I use computer simulation and morphological covariance matrices from the two morphs in a marine snail hybrid zone to show how the proposed procedure can be used to measure the contribution of the matrices orientation and shape to the overall differentiation.

**Results:**

I show how this procedure can detect even modest differences between matrices calculated with moderately sized samples, and how it can be used as the basis for more detailed analyses of the nature of these differences.

**Conclusions:**

The new procedure constitutes a useful resource for the comparison of covariance matrices. It could fill the gap between procedures resulting in a single, overall measure of differentiation, and analytical methods based on multiple model comparison not providing such a measure.

## Background

Covariance matrices are key tools in the study of the genetics and evolution of quantitative traits. The **G** matrix, containing the additive genetic variances and covariances for a set of characters, summarizes the genetic architecture of traits and determines their short-term response to multivariate selection along with the constraints this response will face. The more easily estimated matrix of phenotypic variances and covariances **P** can be used as a surrogate for **G**, especially in the case of high heritability morphological characters [1-4]. Comparisons between covariance matrices are carried out in the study of a wide array of evolutionary problems, such as the stability of **G** in the presence of selection and drift [5-7], the role of genetic constraints on determining evolutionary trajectories in adaptive radiations [8], the response of genetic architecture to environmental heterogeneity [9], the evolution of phenotypic integration [4,10], multi-character phenotypic plasticity [11] and sexual dimorphism [12,13].

Several methods for the comparison of covariance matrices are available (reviewed in [14]). They range from the most mathematically sophisticated, such as those using maximum likelihood [15] or Bayesian frameworks [16], to simple methods that are useful for exploratory analyses and are not dependent on distributional assumptions. The simplest methods [17-20] are based on pair wise comparisons of the matrices’ elements, so that they typically ignore the lack of independence between these values, cannot detect proportionality between matrices, and consider two matrices only. More recent procedures, also using the matrix elements [21], take into account these elements’ lack of independence and permit the joint consideration of several matrices, making it possible to study the contribution of identified external factors to the magnitude of the differentiation.

Other simple procedures [22,23] are based on comparisons between vectors resulting from the product of the studied matrices and sets of test vectors, their rationale being that similar matrices would produce similar results when multiplied by the same sets of vectors. However, most of these procedures result in a single measure of the divergence between matrices that does not provide information about the nature of this divergence. Such information is provided by CPCA (Common Principal Components Analysis [24]), which uses the Flury [25] hierarchy, a maximum likelihood-based procedure to compare the structure of two or more matrices and sequentially test if the matrices are “unrelated” (have no eigenvectors in common); if they have some number of eigenvectors in common, if they are proportional (have all their eigenvectors in common and their eigenvalues keep the same proportions), and finally if they are equal (have all eigenvectors and eigenvalues in common). Then it determines which of these descriptions best fits to the relationship between the matrices’ structures.

Among the limitations of CPCA are, first, that it is based on the assumption of multivariate normality, and second, that it results in categorical, not continuously varying measures of matrix similarity [26]. The CPCA consists in a series of yes/no comparisons between ordered eigenvectors, which allow testing a full series of hypotheses about the relationship between matrices in a hierarchical way, but idoes not have an associated parameter measuring the degree of similarity, relying only on the results of the significance tests. This limitation can be serious in some situations. Two matrices are declared as “unrelated” when that is the best fit of all null hypotheses tested, but this result does not preclude the existence of any similarities between them [14]. In fact, the procedure may declare two matrices as “unrelated” when many data are available and there is great power to detect differences, even if these differences are trivial from a biological point of view [26,27]. Thus, there is no simple relationship between matrix similarities measured by CPCA and other matrix comparison procedures [27].

In the present work I propose a new, simple and distribution-free procedure for the exploration of differences between covariance matrices that, in addition to providing a single and continuously varying measure of matrix differentiation, makes it possible to analyse this measure in terms of the contributions of differences in matrix orientation and shape. I use both computer simulation and **P** matrices corresponding to snail morphological measures to compare this procedure with some widely used alternatives. I show that the new procedure has power similar or better than that of the simpler methods, and how it can be used as the basis for more detailed analyses of the nature of the found differences.

### Pairwise matrix comparison

**Figure 1:**
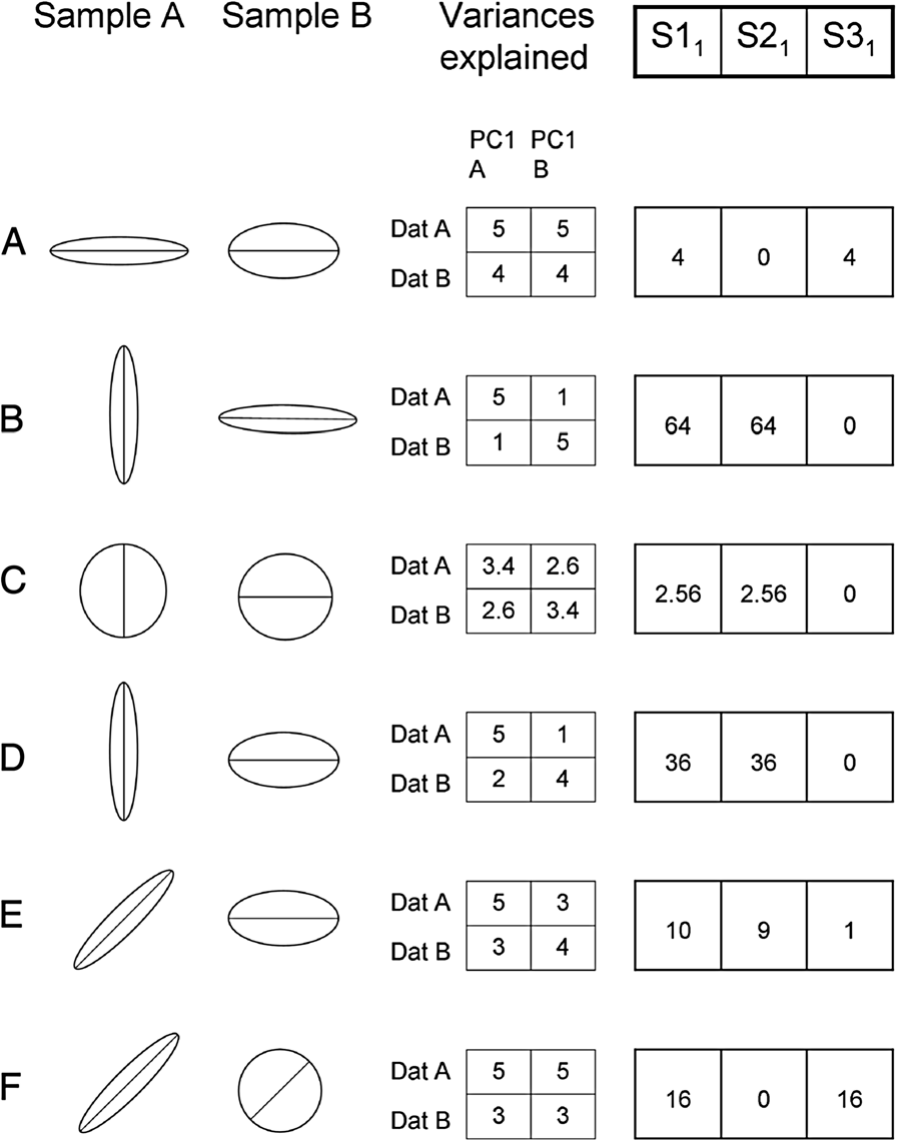
**Contributions (S1**_**1**_**, S2**_**1**_**, S3**_**1**_**) of the first eigenvectors of two sample matrices to the three sums used to measure the differentiation between these matrices in six hypothetical two-variable situations differing in matrices’ shape and orientation. **The ellipse axes’ lengths in the graphics represent the magnitude of the eigenvalues and the orientation of the eigenvectors in the two samples. The straight lines mark the first eigenvectors. The tables in the middle column contain the variances explained by the first eigenvectors obtained in each sample when calculated in the two data sets. Details about the generation of the used matrices are given in Appendix 2.

The rationale for the comparison procedure is that, when the covariance matrices of two data samples are similar, the eigenvectors obtained in a principal component analysis of any of them will explain similar amounts of variation in both samples. The degree of similarity can be measured by calculating in each sample the individuals’ values and variances for the eigenvectors obtained in the other sample. Given that **D**_**1**_ and **D**_**2**_ are the matrices with the characters’ measures in the two samples and **X**_**1**_ and **X**_**2**_ the matrices containing in their columns the eigenvectors of these samples’ covariance matrices, the variances of the columns of the products **D**_**1**_**X**_**1**_ and **D**_**2**_**X**_**2**_ are the corresponding eigenvalues, i.e., the amounts of variance explained by the original eigenvectors, and those of **D**_**1**_**X**_**2**_ and **D**_**2**_**X**_**1**_, the amounts of variance explained by the eigenvectors from the compared sample. Thus, for each of the *n* (number of variables measured) pairs of eigenvectors obtained in the analysis of the two samples it is possible to calculate v_i11_, v_i12_, v_i21_, and v_i22_, where v_i11_ is the amount of sample 1 total variance explained by eigenvector i from sample 1, v_i12_ the amount of sample 1 total variance explained when applying eigenvector i from sample 2, and so on. These n sets of four values are the basic items to measure the similarity in covariance between samples. I define three sums:

$$S1=2\sum_{i=1}^{n}{\left(v_{i11}-v_{i21}\right)}^{2}+{\left(v_{i12}-v_{i22}\right)}^{2}$$

$$S2=\sum_{i=1}^{n}{\left[\left(v_{i11}+v_{i22}\right)-\left(v_{i12}+v_{i21}\right)\right]}^{2}$$

$$S3=\sum_{i=1}^{n}{\left[\left(v_{i11}+v_{i12}\right)-\left(v_{i21}+v_{i22}\right)\right]}^{2}$$

where S1 is a general measure of differentiation depending on the ability of the eigenvectors from each sample to explain the variation in the other sample; S2 is a measure of the contribution of between-matrix differences in orientation (i.e., differences in orientation between eigenvectors in the same ordinal position in the two matrices) to S1, and S3 that of differences in shape (i.e., differences in the proportion of total variance explained by eigenvectors in the same ordinal position in the two matrices). It can be shown (Appendix 1) that S1 = S2 + S3. Figure 1 shows a graphical interpretation of these statistics. In this figure, row A shows that S3 is the only non-zero component of S1 when the matrices differ in shape but not in orientation, and row B that S2 is the only non-zero component when the matrices differ in orientation but not in shape. Note however that S2 and S3 do not measure changes in orientation or shape as such. What these statistics measure are the consequences of changes in orientation and shape on the more functionally relevant proportions of variance explained, i.e.,. on the amount of variation available in each multidimensional direction. The effects of these changes are not independent. The comparison between Figure 1 rows B and C shows that the impact of changes in orientation on the proportion of variance explained by the eigenvectors of the reciprocal matrix depends on the matrices’ shape. The effect is larger for more “elongate” matrices. Similarly, the comparison between rows A, D and E shows that the effect of changes in shape depends on the matrices’ orientation, and that changes in matrix shape become irrelevant (in this two-variables example) when the first eigenvectors of the two matrices are perpendicular (row D). Finally, row F shows that there are no differences in orientation when one of the matrices is perfectly “round” (i.e., all its eigenvectors explain the same amount of total variance). Despite this complexity, and as shown in the practical application made below, statistics S2 and S3 may be very useful to analyse the nature of the differentiation between two covariance matrices.

The S statistics are easier to compare between studies if they are made to vary between zero and one by making them relative to their maximum possible value. This maximum would occur in the extreme situation in which single eigenvectors explain all variation in each of the compared samples, and the eigenvectors of the two samples are orthogonal. In that case, S1 is equal to 2 times the sum of twice the square of the total variance of the first sample and twice the square of the total variance of the second sample. When the variances explained by each eigenvector are expressed as proportions of the total variance, so that the sum of all the proportions is equal to one, the maximum possible value for S1 is equal to eight. In the computer simulation and real data example shown in this article, the explained variances are expressed as proportions, and the S1, S2 and S3 statistics divided by eight so that they could vary between zero and one.

**Figure 2:**
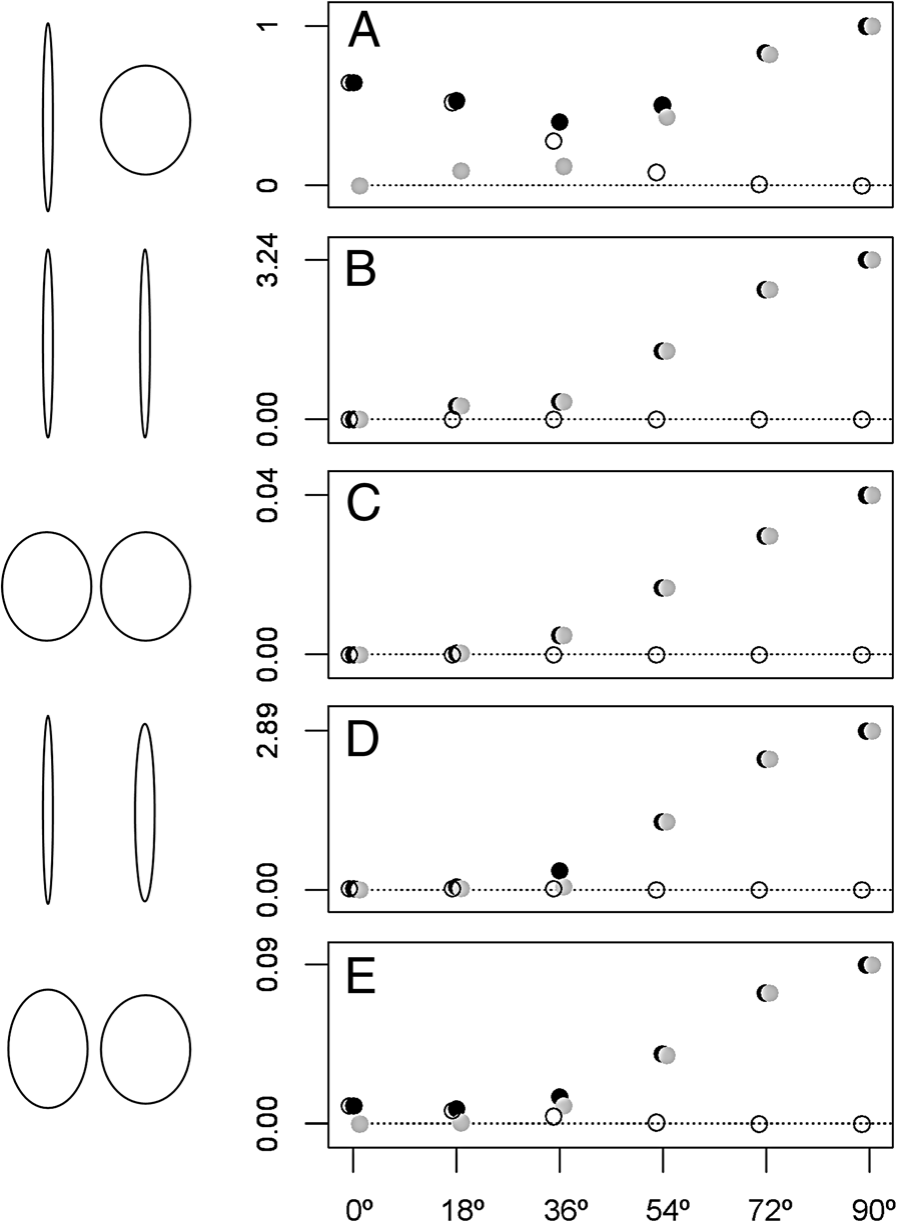
**S1, S2 and S3 statistics values (y axis; black, grey and white points respectively; they are slightly displaced for clarity) in five matrix-shape differences and six relative orientations of the matrices’ first eigenvectors, from zero to 90º (x axis). A**) two matrices with very different shape, one with eigenvalues equal to 95 and 5% of total variance and the other with eigenvalues equal to 55 and 45% of total variance; **B**) two same-shape “elongate” matrices, both with eigenvalues explaining 95 and 5% of total variance; **C**) two same-shape “rounded” matrices both with eigenvalues explaining 55 and 45% of total variance; **D**) two “elongate” matrices with slightly different shapes, one with eigenvalues explaining 95 and 5% of total variance and the other, 90 and 10% of total variance; **E**) two “rounded” matrices with slightly different shapes, one with eigenvalues explaining 60 and 40% of total variance and the other, 55 and 45% of total variance. Matrices are schematically represented at left in a zero degrees relative orientation, with ellipses’ axes equal to the matrices’ eigenvalues. Note that the scale varies between plots.

Figure 2 shows the responses of the three S statistics in a wide range of two-variable situations. S2 is not sensible to changes affecting only matrix shape (A through E, zero degrees case) and S3 is not sensible to changes affecting only matrix orientation (B and C, all relative orientations). Also, and as explained above, the influence of changes in orientation on the proportion of variance explained by the eigenvectors of the compared matrix is heavily dependent on matrix shape. It is large for elongate matrices and modest for rounded ones (compare the maximum values attained by S1 and S2 in B and C). Plots B to E show that the effect of changes in orientation is stronger in high angle ranges than in low angle ones. Finally, plot A shows that, in the same way as the effect of orientation depends on matrix shape, the effect of shape depends on orientation. When matrices differ both in shape and orientation, there is an intermediate angle that minimizes their differences in total variance explained. In addition, when the matrices’ first eigenvectors become perpendicular to each other (far right of all plots), any differences are detected as changes in orientation. These observations emphasise the point made above that what the S statistics measure are differences in the variance explained by two matrices’ eigenvector sets, and therefore in amount of variation in every direction of the multivariate space in the matrices’ original samples. The statistics also measure the influence of matrix shape and orientation on that differentiation, but not differences in shape and orientation directly.

## Results

I contrasted the results obtained with the proposed procedure with those from other widely used ones, namely CPCA and two simpler procedures providing single measures of matrix differentiation: one, the Random Skewers, based on products with test vectors, and the other, the T method, based on the comparison of matrix elements (see Methods). I followed two approaches. First, I studied the procedures’ power and Type I error through computer simulations that considered covariance matrices differing in shape, orientation or both, in different number of variables and sample size situations. Second, I compared their ability to detect differences between covariance matrices of shell measures from different morphs and populations of the seashore snail *Littorina saxatilis*.

### Computer simulation

**Figure 3:**
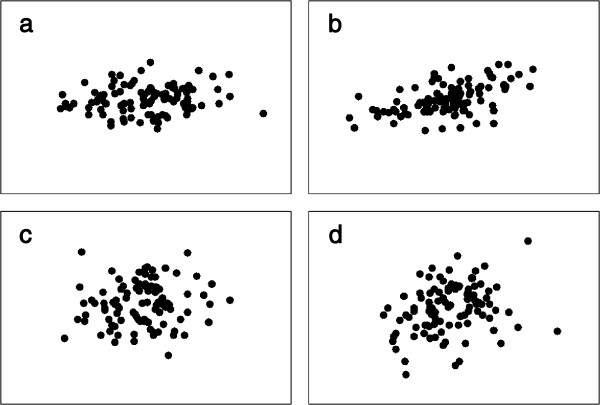
**Examples of population samples used in the simulations (two variables case, size = 100): (a) from the reference population, (b) from the population resulting in a covariance matrix with changed orientation, (c) from the population resulting in a covariance matrix with changed shape, and (d) from the population resulting in a covariance matrix with both orientation and shape changed. **Each axis in the graphs corresponds to one of the two variables.

**Figure 4:**
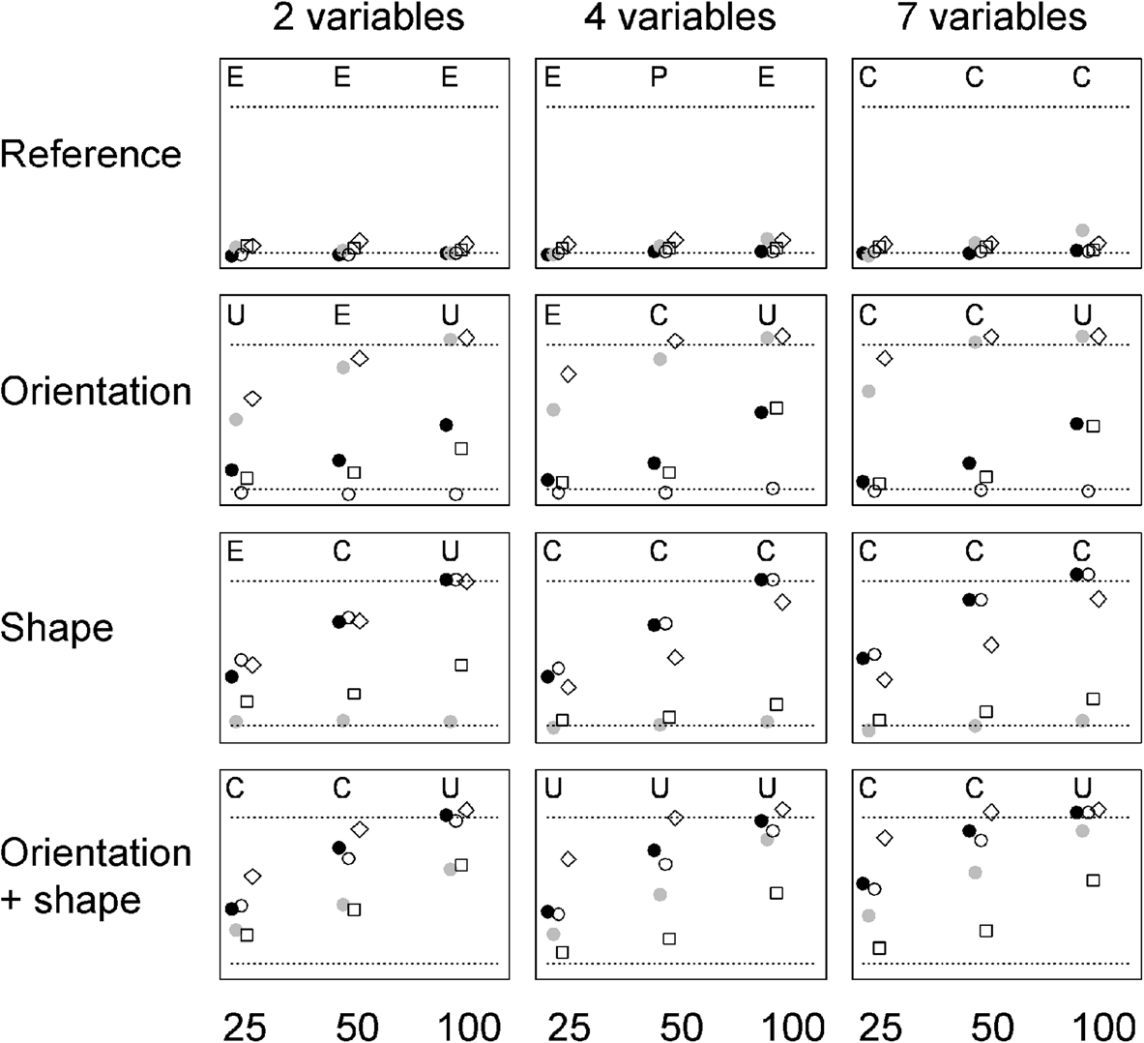
**Proportions (from 0 to 100%; the lower and upper dotted lines mark the 5 and 95% respectively) of simulation replicates in which a difference between covariance matrices was found by the S1, S2, S3 (black, grey and white circles), RS (rhombs) and T method (squares) in comparisons involving matrices of samples taken from the same reference population (reference) or one from the reference population and another from a population resulting in matrices with altered orientation(orientation) or with altered shape (shape) or both (orientation + shape) in situations involving 2, 4 or 7 variables and sample sizes of 25, 50 or 100 individuals. **The sign positions in each sample size were slightly displaced to improve clarity. The top of each graph shows the results of CPC-based comparisons of two samples taken at random from each of the two populations considered in that graph (E: equal, P: proportional, C: CPC result, meaning that all eigenvectors were common but the matrices were not proportional –i.e., same orientation but differences in shape- and U: unrelated matrices). Note: the CPC program considers the possibility that only a subset of eigenvectors are in common, but that result was never found in these simulations.

Figure 3 shows two-variable examples of the four kinds of datasets used in the simulations. The simulation results (Figure 4) show that S2, the T method and RS tended to produce type I errors with frequencies greater than 5% (Figure 4). The average proportions of false positives (calculated across the first row of graphs of the figure, in which a sample of the reference population is compared with another sample for the same population) for the three statistics were 0.096, 0.077 and 0.108, respectively. As expected, the statistic sensitive to changes in orientation, S2, found similar results for comparisons involving matrices diverging only in shape and for comparisons involving matrices from samples of the same population. This was also the case for S3, the statistic sensitive to changes in shape, when applied to populations differing only in orientation. The RS procedure tended to have at least as much power as the most powerful S statistic for the detection of differences in orientation. In fact, it could have been more powerful than the S statistics in the four cases involving changes in orientation, samples of size 100 and more than two variables, because it was able to detect differences in all replicates of these comparisons. In any case, RS seemed to be relatively better for small samples (compare results for sample size 25 with those for sample size 50). However, it was worse than S1 and S3 for the detection of changes in shape. The T method tended to be the least powerful in all situations.

Figure 4 shows also the results obtained with CPCA. These results cannot be directly compared with those of the remaining methods because they were based on a single replicate instead of an average of replicates for each case. However, the results suggest that CPCA could produce an excess of false positives as the number of variables increases (it declared as not equal the matrices from the same population in the seven variables cases). In these simulations, CPCA should declare the matrices as “unrelated” when they differed in orientation, because in that case they would not share any eigenvector in common and as “CPC”, i.e., sharing all eigenvectors but being not proportional when the matrices differed in shape. The procedure tended to correctly detect changes in shape even for small sample sizes, except for the two variables case. Its performance tended to be lower in the detection of changes in orientation in the smaller sized samples. Overall, CPCA tended to agree with the S and RS procedures for the largest (100) sample size.

### *Littorina* data

**Figure 5:**
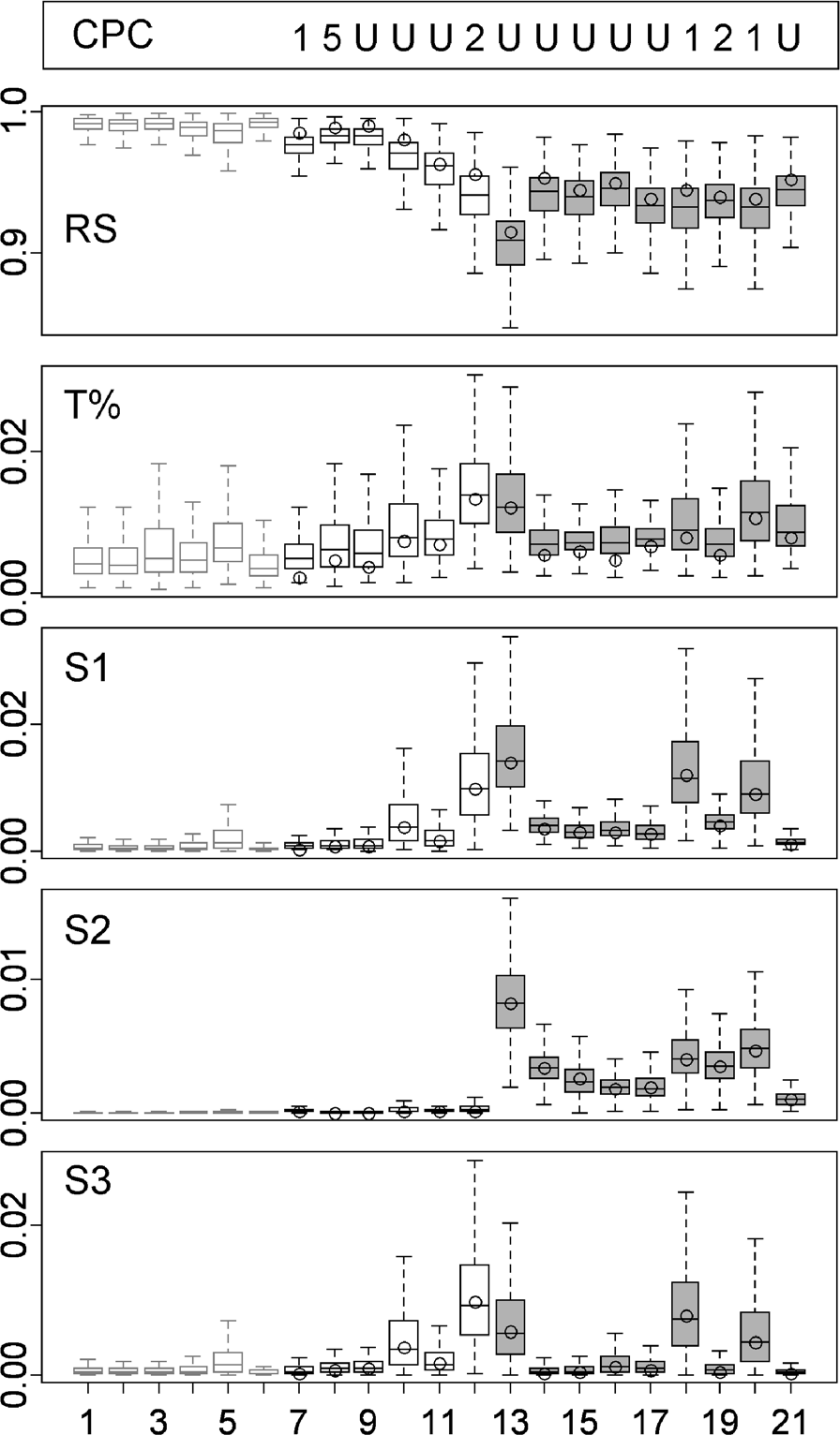
**CPC results and bootstrap distributions for five statistics to compare *Littorina *data covariance matrices in comparisons within sample (grey-lined boxplots; 1 to 3, Rbs; 4 to 6, Sus), between locations within morph (black-lined boxplots; 7 to 9, between Rbs; 10 to 12, between Sus) and between morphs (grey-filled boxplots; 13 to 18, between morphs of different locations; 19 to 21, between morphs of the same location). **The T% values were divided by 100 to make them comparable with the other statistics. The CPC box shows the number of common principal components; U: unrelated. No CPC analysis was done for the comparisons within samples. Plots do not include outliers. Circles mark the observed values for the statistics. No observed values are printed in the case of within sample comparisons (i.e., of matrices with themselves) because they were always equal to one for the RS and equal to zero for the other statistics. Comparison codes: 1, Rb1-Rb1; 2, Rb2-Rb2; 3, Rb3-Rb3; 4, Su1-Su1; 5, Su2-Su2; 6, Su3-Su3; 7, Rb1-Rb2; 8 Rb1-Rb3; 9, Rb2-Rb3; 10, Su1-Su2; 11, Su1-Su3; 12, Su2-Su3; 13, Rb1-Su2; 14, Rb2-Su1; 15, Rb1-Su3; 16, Rb3-Su1; 17, Rb2-Su3; 18, Rb3-Su2; 19, Rb1-Su1; 20, Rb2-Su2; 21, Rb3-Su3.

The eigenvectors and eigenvalues of the six samples’ covariance matrices are shown in Table 1. The first eigenvectors always had coefficients of the same sign, as expected for size descriptors [28], and were similar for all matrices. The remaining eigenvectors included coefficients of contrasting signs, as expected if they were measuring variation in shell shape. The bootstrap distributions of the RS, T%, S1, S2 and S3 statistics for 21 comparisons are shown in Figure 5. Not unexpectedly, some basic common trends were observed in all these statistics. Matrices in within-sample comparisons tended to be found more similar than those in within morph comparisons, and these, more than matrices in between morph comparisons. Also, particular between-samples comparisons resulting in large differences when applying a procedure tended to do the same in the remaining procedures. The T method was, of those shown in Figure 5, the least sensitive to matrix differences. In the case of the RS, S1 and S2 statistics, the bootstrap distributions of the within sample and between morphs comparisons had little overlap, showing that these statistics can easily detect rather modest differences (all observed values for the S-statistics measures of differentiation were lower than 0.02 for a range of possible values from 0 to 1, and those for the RS statistic-measures of similarity were higher than 0.9 for a range of possible values from 1 to 0) between matrices using samples of moderate size. The RS method tended to provide a rather definite separation among the results of four kinds of comparisons: within samples, within Rbs, within Sus and between morphs. The profile of the S statistics’ comparisons was rather different from that obtained with the RS procedure, indicating that the two methods are not equivalent and that they consider matrices’ properties in different ways.

**Table 1:** Eigenvector analysis

***Sample***	***EG1***	***EG2***	***EG3***	***EG4***	***EG5***	***EG6***	***EG7***
Rb loc 1							
	0.349	0.178	0.046	0.428	−0.049	−0.596	0.551
	0.297	0.464	0.231	0.492	0.139	0.614	−0.064
	0.323	0.350	0.209	−0.711	0.396	0.008	0.260
	0.365	0.010	−0.033	−0.260	−0.870	0.170	0.047
	0.427	−0.068	−0.867	0.011	0.219	0.107	−0.049
	0.499	−0.765	0.354	0.041	0.127	0.146	0.039
	0.347	0.176	0.152	0.011	0.026	−0.454	−0.790
	*21e-2*	*28e-3*	*62e-4*	*11e-4*	*76e-5*	*28e-5*	*18e-5*
	**85.48**	**11.14**	**2.48**	**0.42**	**0.30**	**0.11**	**0.07**
Rb loc 2							
	0.349	0.125	0.133	0.432	−0.335	−0.568	0.473
	0.306	0.305	0.323	0.464	0.235	0.648	0.122
	0.314	0.226	0.339	−0.567	0.551	−0.259	0.208
	0.377	0.124	0.137	−0.490	−0.700	0.304	−0.032
	0.447	0.250	−0.843	−0.020	0.154	0.050	0.027
	0.471	−0.865	0.023	0.027	0.125	0.098	0.058
	0.357	0.185	0.127	0.005	−0.001	−0.748	−0.512
	*21e-2*	*27e-3*	*91e-4*	*12e-4*	*71e-5*	*49e-5*	*11e-5*
	**84.24**	**11.00**	**3.66**	**0.50**	**0.29**	**0.20**	**0.11**
Rb loc 3							
	0.362	0.143	0.124	0.481	−0.094	−0.599	0.483
	0.306	0.411	0.334	0.406	0.020	0.678	0.004
	0.317	0.284	0.209	−0.616	0.567	−0.068	0.264
	0.363	0.099	0.060	−0.464	−0.797	0.048	0.049
	0.417	0.131	−0.881	0.062	0.111	0.126	−0.008
	0.489	−0.828	0.152	0.037	0.124	0.180	0.050
	0.360	0.129	0.161	0.060	0.085	−0.354	−0.832
	*18e-2*	*17e-3*	*87e-4*	*79e-5*	*49e-5*	*21e-5*	*18e-5*
	**83.44**	**12.85**	**2.20**	**0.93**	**0.40**	**0.13**	**0.05**
Su loc 1							
	0.325	0.176	0.147	0.272	0.196	−0.375	0.767
	0.245	0.291	0.395	0.414	0.379	0.585	−0.204
	0.288	0.309	0.346	−0.198	−0.788	0.178	0.100
	0.360	0.120	0.063	−0.817	0.426	0.055	0.016
	0.493	−0.838	0.200	0.076	−0.071	0.056	−0.037
	0.523	0.154	−0.796	0.152	−0.099	0.184	−0.043
	0.324	0.221	0.158	0.138	0.014	−0.668	−0.597
	*20e-2*	*25e-3*	*14e-3*	*15e-4*	*99e-5*	*27e-5*	*15e-5*
	**82.97**	**10.07**	**5.76**	**0.62**	**0.40**	**0.11**	**0.06**
Su loc 2							
	0.343	0.101	0.186	0.305	0.2812	−0.529	0.620
	0.277	0.128	0.474	0.356	0.224	0.711	0.014
	0.281	0.181	0.365	−0.119	−0.853	−0.059	0.100
	0.365	0.108	0.145	−0.848	0.327	0.062	0.060
							
	0.469	−0.866	−0.126	0.039	−0.094	0.066	0.000
	0.506	0.408	−0.723	0.125	−0.086	0.176	−0.001
	0.343	0.114	0.217	0.170	0.144	−0.414	−0.775
	*16e-2*	*33e-3*	*13e-3*	*17e-4*	*11e-4*	*35e-5*	*16e-5*
	**76.48**	**15.88**	**6.06**	**0.80**	**0.54**	**0.17**	**0.08**
Su loc 3							
	0.338	0.124	0.204	0.308	−0.222	−0.378	−0.736
	0.255	0.192	0.451	0.459	−0.202	0.634	0.202
	0.282	0.200	0.356	−0.344	0.767	0.124	−0.176
	0.322	0.126	0.152	−0.737	−0.555	0.070	0.035
	0.425	−0.899	0.073	0.015	0.051	0.044	0.031
	0.594	0.237	−0.739	0.097	0.089	0.161	0.038
	0.322	0.166	0.231	0.153	0.044	−0.638	0.618
	*24e-2*	*17e-3*	*14e-3*	*16e-4*	*13e-4*	*24e-5*	*96e-6*
	**87.32**	**6.74**	**5.15**	**0.38**	**0.27**	**0.08**	**0.06**

The columns show each eigenvector’s coefficients for the variables measured in each sample, along with the corresponding absolute eigenvalues (cursive) and the same eigenvalues as percentages of the total variance in the sample (bold).

**Figure 6:**
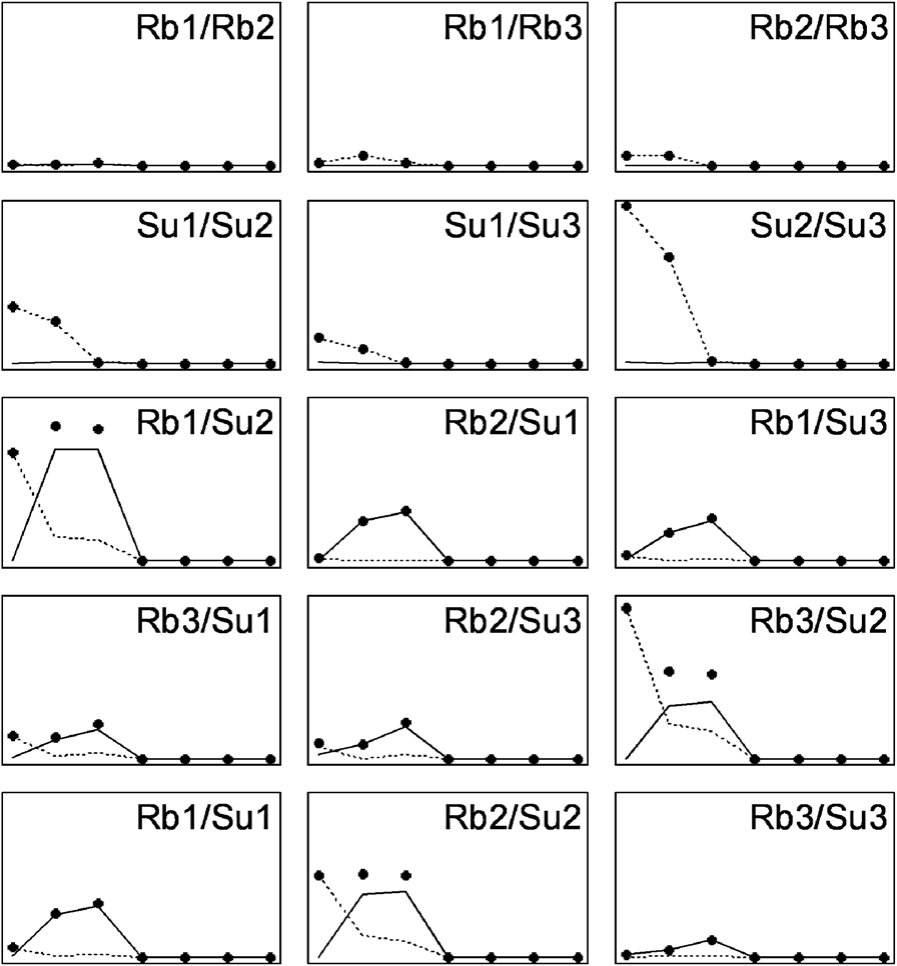
**Representation of the contribution (vertical axes) of each of the seven eigenvector pairs (1 to 7 from left to right in the horizontal axis) to the S1 (black points), S2 (solid lines) and S3 (dashed lines) statistics in each comparison between samples. **All graphs are drawn to the same scale (minimum 0, maximum 0.0058) to ease comparison.

At least in the particular example analyzed here, differences related with matrix shape had the largest weight in the overall measure of differentiation S1, as the comparison results profiles of S1 and S3 were the most similar. The statistic S3 found large differences both between morphs and within the Su morph. The largest differences for S3 corresponded always to comparisons involving the matrix of the Sus from location 2, i.e., comparisons 10, 12, 13, 18 and 20 in Figure 4. This suggested that the shape of this matrix had some particularities, and in fact Table 1 shows that the first and second eigenvalues for this sample were unusually small and large, respectively. A more detailed analysis of the S statistics (Figure 6) was consistent with this interpretation: the largest contributions to S3 were made by the first eigenvector and occurred in comparisons involving the Su2 sample.

**Figure 7:**
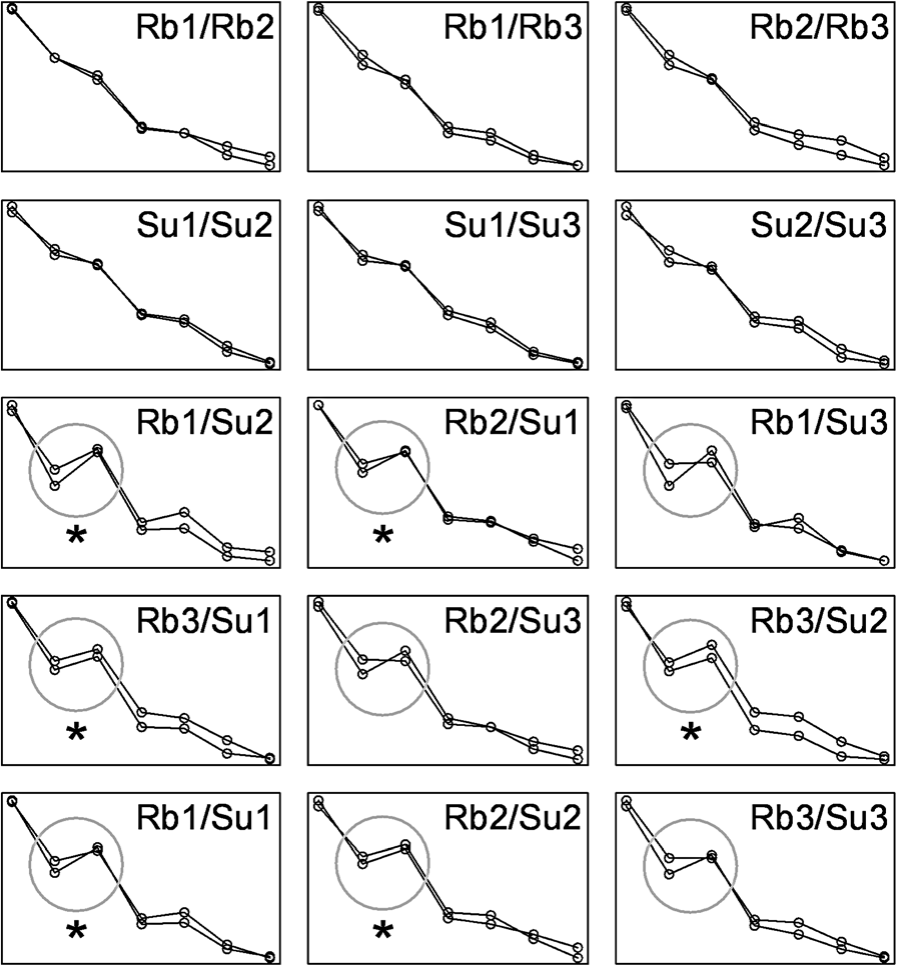
**Proportions of the total variance (vertical axis; log-transformed for clarity of representation) of each sample explained by the eigenvectors (1 to 7 from left to right in the horizontal axis) obtained in the analysis of the reciprocal sample in each between-samples comparison. **The gray circles mark the increases in variance explained by higher order reciprocal eigenvectors. The asterisks correspond to bootstrap tests of the change in proportion of variance explained (average of the two reciprocal comparisons) by successive eigenvectors. They mark changes in which the 97.5 percentile of the bootstrapped distribution was negative (i.e., the third reciprocal eigenvector explained more variance than the second).

The statistic S2 found the most striking contrast between kinds of comparisons in Figure 5, differences between morphs being clearly the largest. Thus, a large proportion of the differentiation between the two morphs’ covariance patterns was due to a change in matrix orientation not present in the comparisons within morphs. Figure 5 shows that the largest contributions to S2 were made by the second and third eigenvectors. The examination of these eigenvectors’ coefficients in Table 1 shows that the second eigenvector from the Rbs was similar to the third eigenvector from the Sus, and vice versa. The second eigenvector from the Rbs and the third from the Sus could be roughly described as a contrast of measure 6 against the rest, and the third eigenvector of the Rbs and the second from the Sus as a contrast of measure five against the rest. Thus, the two morphs’ eigenvectors had very similar structure, but the proportion of variance explained by these eigenvectors was clearly different, to the extent that the ranks of the second and third eigenvectors in one morph got reversed in the other morph. This change in matrix orientation introduced large differences in the proportion of variance explained by the reciprocal eigenvectors. This is illustrated in Figure 7, representing the amount of variance explained in a sample by the eigenvectors of the compared sample (v_i12_ and v_i21_ of the S expressions, see above). It can be seen that the differentiation between the Rb and Su matrices is related to a reversal in the variances explained by the second and third eigenvectors. In both morphs, the third eigenvector from the reciprocal morph explains more variation than the second reciprocal eigenvector. The figure shows also that the lowest differentiation was in comparisons involving sample Su3. Again, the inspection of Table 1 results in an easy interpretation: the second and third eigenvalues were very similar in this sample (6.74 and 5.15%), so that reversing the order of the corresponding eigenvectors (as done to a good extent in the comparisons with Rb samples) had very slight effects on the proportion of variance explained. Note in Figure 7 that one of the segments in the circles is nearly horizontal for the comparisons involving sample Su3. Not all results had a straightforward interpretation in terms of eigenvector coefficients. For example, Figure 5 shows that the S-statistics comparisons between the Rb and Su samples from location 3 resulted in markedly lower differentiation than all the remaining between morph comparisons. This shows that the S procedures consider aspects of matrix structure beyond the individual values of eigenvector’s coefficients.

The overall agreement between CPCA and the other procedures found in the simulations was lost in the analysis of *Littorina* data. (upper side of Figure 5). According to the RS, T% and S statistics, the three Rb samples had very similar covariance matrices, but the CPC procedure determined that Rb2 and Rb3 were “unrelated”, and that Rb1 and Rb2 had only one eigenvector in common. The few comparisons finding some eigenvectors in common did not correspond to particularly low measures of differentiation by the other methods. This shows that the CPC analysis did not consider the matrices’ properties in the same way.

## Discussion

The S statistics constitute sensitive tools for the detection of differences between covariance matrices. In the *Littorina* example used here, it was found that the local differentiation was clearly higher for the Su than for the Rb morph. This could be due to lower genetic connectivity for populations of the Su morph, and also to environmental differences between localities. However, the absence of such differences between the Rbs in the same localities would imply that any environmental differences would exist not between whole localities but only between the micro-habitats the Su snails use in the mid-shore. The differences between morphs, and specially those due to changes of orientation in the covariance matrices, were the largest in the analysis, and if not of the same size, they were of the same nature in all locations. Since shell morphology variation is adaptively important in *Littorina*[29], this suggests that these differences in covariance could be relevant for the evolutionary dynamics of the hybrid zone. They could simply result from the environmental differences between the two morphs’ microhabitats within the midshore, but even these environmental differences could have a genetic origin because individuals of the two morphs, even when living within extremely short distances of each other, tend to actively choose microhabitats with different physical characteristics [30].

The computer simulations shown in Figure 4 are limited to measure the power of the considered procedures to detect the consequences of changes in matrix orientation and shape. They show that such differences can be detected even when moderate in magnitude and when sample sizes are not too large, but cannot be taken as complete or definitive comparisons between the procedures. Matrices may differ in many relevant aspects, and different comparison procedures may have different aims and take different aspects into consideration. For example, while the S measures consider the differences in eigenstructure between two matrices, the RS procedure focuses on the related, but not equivalent problem of the differences between the evolutionary responses generated by these matrices. Comparisons would be even more difficult with procedures such as the set of evolvability measures proposed by Hansen and Houle [31], which consider the magnitude of the populations’ responses to different natural selection regimes.

Since the S statistics introduced here simply measure what proportion of variation exists in a given sample along the axis of variation defined by the eigenvectors in the compared sample, they are similar to the RS and T% ones in that they do not compare and are not dependent on the matrices’ sizes. They focus instead on the more interesting differences in matrix shape and orientation. In any case, S statistics-based comparisons could use raw covariance components instead of proportions as in the example shown, so that the results would depend on between-matrix size differences. However, in that case the S statistics would not be able to separate the effect of size from those of other sources of differentiation between matrices. Similarly, the basic version of the T method proposed in [19] reflects the differences in matrix size, as it is based on raw variance components instead of the proportions used by the T% statistic.

Calculating the amount of variance explained by a set of eigenvectors in a given dataset is straightforward in the case of datasets containing the phenotypic measures used to obtain **P** matrices. In the case of **G** matrices, the comparison would have to be based on additive genetic value estimates for individuals or families.

Since the proposed procedure is limited to two-sample comparisons, it cannot be used to make higher order analyses of the divergence among several populations (see [32]). However, and as shown in the *Litttorina* example, it can be useful for the study of evolutionary relevant situations such as hybrid zones. The S measures appear to be similar to the measure of distance between covariance matrices used by Mitteroecker and Bookstein [33], based on the calculation of relative eigenvalues, i.e., the eigenvalues of the product of one matrix premultiplied by the inverse of the second matrix, and therefore on expressing the variances and covariances of one sample relative to those of the other sample. I calculated the correlation of this measure with S1, S2 and S3 in a computer simulation considering the same cases as in Figure 4 and found that, while the measures were clearly related, they were not equivalent. For example, in the simulation of four variables and sample size 50, the correlation across replicates of the Mitteroecker and Bookstein measure with S1, S2 and S3 were: in the comparison of one population with itself, 0.206, 0.399 and 0.140 respectively; in the case of divergence in orientation, 0.396, 0.533 and 0.040^NS^; in the case of divergence in matrix shape, 0.568, 0.281 and 0.554; and in the case of divergence in both orientation and shape, 0.454, 0.572 and 0.341 (all correlations, P < 0.001 except when indicated).

The *Littorina* example supports the view that CPCA might lead to misleading conclusions about the overall similarity between matrices [26], as pairs of matrices found very similar by other procedures were declared as “unrelated” by CPCA, and there was no clear correspondence between the two sets of results. The observed between-morphs reversal in importance of the second and third eigenvectors could play a role in this discrepancy. CPCA is based on a series of paired comparisons between eigenvectors of the same rank. Two matrices may share their axes of variation, but not the amount of variance in each axis. For example, the *ith* eigenvector of one matrix might be the same as the *i+1* eigenvector of the other, and the *i+1* of the first, the same as the *ith* of the second. In that case, the two matrices would have the same eigenvectors, but in a reverse order. A comparison between their *ith* eigenvectors would find them orthogonal, and this would also be the case for their *i+1* eigenvectors. Thus there may be considerable similarity between the two matrices, but this similarity is overlooked by the comparison procedure which finds the paired eigenvectors very different. The CPC software [34] enables users to compare the eigenvectors in any order, but this does not fix this particular limitation, as the order chosen for the two samples must be the same. The T and RS methods, based on matrices’ elements and product vectors, would provide a more balanced measure of similarity in this situation because the differences between these elements and these vectors would not depend on the existence of reversals in eigenvector order per se, but on the magnitude of the differences involved. However they don’t allow further analysis of the pattern of differentiation. The three S statistics are affected by different patterns of divergence, so that their joint use provided a deeper view of the differences between the two morphs’ **P** matrices. The S1 statistic is not dependent on the eigenvectors’ ordering per se because it is based on comparisons within eigenvector, i.e., on the difference between the amount of variance explained by one eigenvector from one sample in the original and reciprocal samples. These differences do not change with eigenvector order. But S2 changes when the order of eigenvectors in one of the samples is reversed (see formulas) because this would be considered as a change in matrix orientation. In case the reversal in eigenvectors’ importance was complete, so that there were no changes in overall shape, S3 would remain unaffected (see the second row in Figure 1). However, the reversal of the second and third eigenvectors between morphs cannot fully explain the disagreement between CPCA and the remaining methods because the results for S2 were not particularly similar to those of CPCA (Figure 5). This suggests that other aspects of covariance matrix structure might control the degree of agreement between different comparison procedures.

## Conclusions

The S-statistics procedure provides a simple and continuously-varying overall measure of differentiation that is distribution free and interpretable in terms of changes in matrix orientation and shape. In addition, it makes it easy to study the contribution of the different eigenvectors to the statistics values, which could provide further details on the nature of the differentiation, as was the case of the *Littorina* example used. This procedure could thus fill the gap between simpler statistics such as T% and RS, and more analytical methods like CPCA or Bayesian MCMC. The S-statistics procedure is not based on a formal analysis of matrices’ properties. Instead, it could serve for a simple and fast exploration of the magnitude and nature of the differentiation.

## Methods

### Compared procedures

The random skewers (RS) procedure was proposed by Cheverud [22]. Random selection vectors are generated by sampling selection coefficient values for each of the measured variables from a uniform distribution between 0 and 1. Then they are assigned positive or negative signs with 0.5 probabilities, assembled in selection vectors *β* and the sum of their squared coefficients made equal to one. Next, the responses corresponding to these selection vectors are calculated by multiplying them by each of the compared covariance matrices. If the compared matrices **A** and **B** are similar, the magnitude and direction of their responses to the same selection vector *β*_*i*_ will be similar. The correlation between the two response vectors **A***β*_*i*_ and **B***β*_*i*_ is calculated as

$$corr\left(\text{A}\beta_{i},\text{B}\beta_{i}\right)=\frac{\left(\text{A}\beta_{i}\right)'\left(\text{B}\beta_{i}\right)}{\sqrt{\left[\left(\text{A}\beta_{i}\right)'\left(\text{A}\beta_{i}\right)][\left(\text{B}\beta_{i}\right)'\left(\text{B}\beta_{i}\right)\right]}}$$

and the measure of similarity between matrices as the average correlation for all vectors.

In the T method [19] the differentiation between two matrices is measured as the sum of the absolute differences between the two matrices’ elements. In particular, I used the T% statistic, in which the sum is made relative to the average size of the elements in the two matrices [20]:

$$T\%=\frac{\sum_{i=1}^{c}\left|M_{i1},-,M_{i2}\right|/c}{\left({\overset{―}{M}}_{1}-{\overset{―}{M}}_{2}\right)/2}100$$

where *c* is the number of nonredundant elements in the matrices, M_i1_ and M_i2_ are such elements in the two matrices, and $M_{1}^{―}$ and $M_{2}^{―}$ their averages. The proportional nature of T% makes the comparison between matrices of different sizes easier. It is unreliable when there are both positive and negative elements in any of the two matrices [35], but this was not the case in the examples used in this work to compare the different methods’ performances.

Finally, I used the CPC (Common Principal Components) software of Phillips and Arnold [34] carrying out CPCA. I chose the “step-up” option, in which the likelihood of any model is tested against the likelihood of the next lowest model in the hierarchy, and used the Akaike Information Criterion to select the best description.

### Simulations

The simulations compared pairs of samples of individuals differing in the shape and orientation of their covariance matrices for the measured variables. All variables considered in the simulations had two normally-distributed components, one (*s*) specific for that variable and the other (*c*) common to all variables. The value for variable *i* measured on individual *j* in a given sample was generated as:

$$y_{\text{ij}}=s_{\text{ij}}+k_{\text{i}}c_{\text{j}}$$

In each sample, matrix orientation was controlled by the relative contribution (fixed within sample) *k*_i_ of the common component to each variable’s value, and matrix shape, by the *s*_i_ variances. Four kinds of sample matrix comparisons were made: between samples taken at random from the same population, between samples from populations whose covariance matrices differed in orientation, whose matrices differed in shape, and whose matrices differed in both orientation and shape. One sample was taken at random from each of the two populations compared in each simulation case, and their covariance matrices and comparison statistics calculated. The observed value of each statistic was compared with the distribution obtained by comparing 50 pairs of resamples of the same size taken from the first sample, and with that obtained by comparing 50 pairs of resamples of the same size taken from the second sample. If the observed value was greater than these 100 resampled values, I concluded that the statistic found differences between the two samples’ matrices. This process was repeated 1000 times for each simulation case. I considered three sample sizes, 25, 50 and 100, and three numbers of variables, 2, 4 and 7 (the number of variables in the *Littorina* data). The particular changes in shape and orientation considered in each simulation case were chosen so that at least one of the three S statistics had nearly (but not exactly) 100% power to detect differences. The list of sample parameters used in the simulations is shown in Appendix 3.

### *Littorina* data

**Figure 8:**
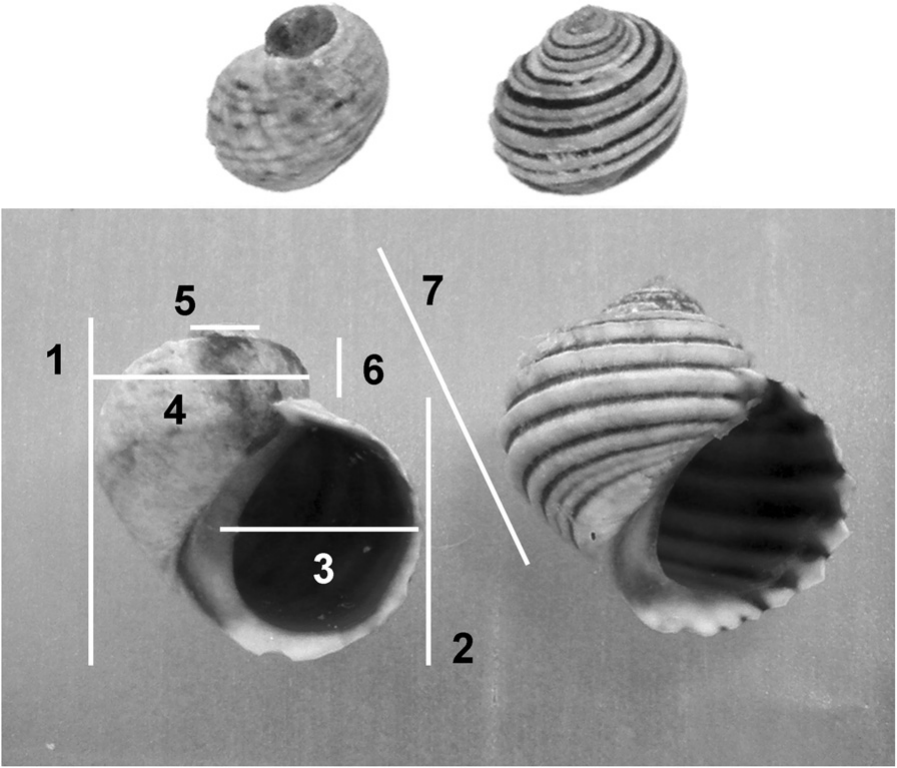
**Back and opercular view of shells of the lower-shore Su (left) and upper shore Rb (right) morphs of *Littorina saxatilis *form the Galician coasts. **The seven measures used are shown on the Su shell. Note: Rb snails are on average larger than Sus; shells of similar sizes were chosen to ease comparison on the image.

I assayed the proposed matrix comparison method on six sets of shell morphology data from the two morphs (Rb and Su) of the marine snail *Littorina saxatilis*, taken in the Galician (NW Iberia) shores. The two morphs occupy different shore levels but form a hybrid zone in the midshore (see [36] for a detailed description of the hybrid zone). Seven shell measures were available (Figure 8, see [37] for more information), all of them taken on pure morph snails from the midshore (only a proportion of the individuals in this area are hybrids) of three locations (Centinela 42º04’N, 8º53’W; Corrubedo, 42º32’N, 9º20’W and Senin, .42º02’N, 8º53’W) and log transformed. The numbers of Rb and Su individuals from each location were 160 and 135, 150 and 112, and 123 and 111. I made three kinds of sample comparisons. First, I compared each sample with itself by generating pairs of pseudo-samples and their covariance matrices by resampling with replication in that sample’s dataset. Comparing samples with themselves provides a reference of maximum possible similarity, useful to interpret the similarities found between different samples. This is the “matrix repeatability” approach used in [26]. Second, I compared matrices from the same morph and different locations; and third, from different morphs. I obtained confidence distributions for the RS, T% , S1, S2 and S3 statistics by bootstrapping (999 replicates) the observations within each of the compared data sets, and obtained the statistics’ values for random pairs of the corresponding covariance matrix. This procedure was more demanding in the case of the RS statistic, because its observed values were already averages of correlations with randomized selection vectors (1000 vectors per value in this work).

## Appendix

### Appendix 1

The v_ijk_ values in Table 2 below, representing the variance explained by the *ith* eigenvector from the covariance matrix of sample *k* when applied to the data of sample *j* can be used to show that twice the sum of the squared differences across columns

$$\begin{array}{l} S1_{i}=2\left[{\left(v_{i11}–v_{i21}\right)}^{2}+{\left({\text{v}}_{\text{i}12}–{\text{v}}_{\text{i}22}\right)}^{2}\right]\\ \;=2\left[{{\text{v}}_{\text{i}11}}^{2}+{{\text{v}}_{\text{i}12}}^{2}+{{\text{v}}_{\text{i}21}}^{2}+{{\text{v}}_{\text{i}22}}^{2}–{2\;\text{v}}_{\text{i}11}{\text{v}}_{\text{i}21}–{2\text{v}}_{\text{i}12}{\text{v}}_{\text{i}22}\right] \end{array}$$

Is the sum of the squared difference between diagonals:

$$\begin{array}{l} {\text{S}2}_{\text{i}}={\left[\left({\text{v}}_{\text{i}11}+{\text{v}}_{\text{i}22}\right)-\left({\text{v}}_{\text{i}12}+{\text{v}}_{\text{i}21}\right)\right]}^{2}\\ \;={{\text{v}}_{\text{i}11}}^{2}+{{\text{v}}_{\text{i}12}}^{2}+{{\text{v}}_{\text{i}21}}^{2}\\ \;+{{\text{v}}_{\text{i}22}}^{2}–{2\text{v}}_{\text{i}11}{\text{v}}_{\text{i}12}–{2\;\text{v}}_{\text{i}11}{\text{v}}_{\text{i}21}–{2\text{v}}_{\text{i}22}{\text{v}}_{\text{i}12}–{2\text{v}}_{\text{i}22}{\text{v}}_{\text{i}21}\\ \;+{2\text{v}}_{\text{i}11}{\text{v}}_{\text{i}22}+{2\text{v}}_{\text{i}12}{\text{v}}_{\text{i}21} \end{array}$$

**Table 2:** Amounts of variance explained by th eith eingeinvectors of each compared sample

	**A** ***ith*** **eigenvector**	**B** ***ith*** **eigenvector**
Sample A	v_i11_	v_i12_
Sample B	v_i21_	v_i22_

 and the squared difference between rows:

$$\begin{array}{l} {\text{S}3}_{\text{i}}={\left[\left({\text{v}}_{\text{i}11}+{\text{v}}_{\text{i}12}\right)-\left({\text{v}}_{\text{i}21}+{\text{v}}_{\text{i}22}\right)\right]}^{2}\\ \;={{\text{v}}_{\text{i}11}}^{2}+{{\text{v}}_{\text{i}12}}^{2}+{{\text{v}}_{\text{i}21}}^{2}\\ \;+{{\text{v}}_{\text{i}22}}^{2}–{2\text{v}}_{\text{i}11}{\text{v}}_{\text{i}21}–{2\;\text{v}}_{\text{i}11}{\text{v}}_{\text{i}22}–{2\text{v}}_{\text{i}12}{\text{v}}_{\text{i}21}–{2\text{v}}_{\text{i}12}{\text{v}}_{\text{i}22}\\ \;+{2\text{v}}_{\text{i}11}{\text{v}}_{\text{i}12}+{2\text{v}}_{\text{i}21}{\text{v}}_{\text{i}22} \end{array}$$

So that

$$\begin{array}{l} \text{S}2_{\text{i}}+\text{S}3_{\text{i}}=2[{{\text{v}}_{\text{i}11}}^{2}+{{\text{v}}_{\text{i}12}}^{2}+{{\text{v}}_{\text{i}21}}^{2}+{{\text{v}}_{\text{i}22}}^{2}-2{\text{v}}_{\text{i}11}{\text{v}}_{\text{i}21}\\ \;-2{\text{v}}_{\text{i}12}{\text{v}}_{\text{i}22}]=\text{S}1_{\text{i}} \end{array}$$

and therefore

$$S1=\sum_{i=1}^{n}S1_{i}=\sum_{i=1}^{n}\left(S2_{i}+S3_{i}\right)=S2+S3$$

### Appendix 2

The covariance matrices used to draw plots in Figure 2 corresponded to pairs of variables *y*_*i*_ (*i* = 1, 2) defined as

$$y_{i}=s_{i}+\text{sqrt}\left(z_{i}\right)c$$

where sqrt is the square root. Note that sqrt(*z*) is equivalent to *k* in the main text. The variances of *s* and *c* were:

$$\begin{array}{l} \mathrm{Case}\;\mathrm{First}\;\mathrm{sample}\;\mathrm{Second}\;\mathrm{sample}\\ \;\mathrm{Variance}\;\mathrm{Variance}\\ \;s\;c\;s\;c\\ A:\;0.1\;0.9\;0.9\;0.1\\ B:\;0.1\;0.9\;0.1\;0.9\\ C:\;0.9\;0.1\;0.9\;0.1\\ D:\;0.1\;0.9\;0.2\;0.8\\ E:\;0.8\;0.2\;0.9\;0.1 \end{array}$$

The angles between eigenvector sets were determined by the *z* coefficients in each sample:

$$\begin{array}{lllll} \mathrm{Angle} & \mathrm{First} & \mathrm{sample} & \mathrm{Second} & \mathrm{sample}\\ \; & z_{1} & \;z_{2} & z_{1} & \;z_{2}\\ \;0^{{}^{\circ}}\;: & 2 & \;0 & 2 & \;0\\ \;18^{{}^{\circ}}: & 2 & \;0 & 1.81 & \;0.19\\ \;36^{{}^{\circ}}: & 2 & \;0 & 1.31 & \;0.69\\ \;54^{{}^{\circ}}: & 2 & \;0 & 1.69 & \;0.31\\ \;72^{{}^{\circ}}: & 2 & \;0 & 0.19 & \;1.81\\ \;90^{{}^{\circ}}: & 2 & \;0 & 0 & \;2 \end{array}$$

In this two-variables case, the variance explained by eigenvector *i* from sample *j* on sample *m* was calculated as

$$v_{\mathrm{ijk}}=e_{\mathrm{ij}}’e_{1m}\mathrm{sqrt}\left(V_{1m}\right)+e_{\mathrm{ij}}’e_{2m}\mathrm{sqrt}\left(V_{2m}\right)$$

where **e**_**ij**_ and ***e***_***im***_ are eigenvectors *i* from samples *j* and *m*, V_im_ is eigenvalue *i* from sample *m* and the apostrophe indicates transposition.

### Appendix 3

#### Summary of cases

The following tables show the values for the variances of variables *s* and *c* and the values of coefficient *z* in the expression:

$$y_{\mathrm{ij}}=s_{\mathrm{ij}}+\mathrm{sqrt}\left(z,i\right)c_{j}$$

used to generate the data for variable *i* and individual *j* in the different cases considered in the simulations -sqrt: square root; note that sqrt(*z*) is equivalent to *k* in the main text. Two variables

$$\begin{array}{llll} \mathrm{Case} & \;\mathrm{Variance} & \;z_{i}\left(i=1,2\right)\\ \; & \;s & \;c & \;\\ \mathrm{Reference}\;\mathrm{sample}: & 0.2 & \;0.8 & \;2,0\\ \mathrm{Change}\;\mathrm{Orient}\;: & 0.2 & \;0.8 & \;1.9,0.1\\ \mathrm{Change}\;\mathrm{shape}\;: & 0.5 & \;0.5 & \;2,0\\ \mathrm{Both}\;\mathrm{chages}\;: & 0.5 & \;0.5 & \;1.9,0.1 \end{array}$$

Four variables

$$\begin{array}{llll} \mathrm{Case} & \mathrm{Variance} & \;z_{i}\left(1=1,4\right)\\ \; & \;s & \;c & \;\\ \mathrm{Reference}\;\mathrm{sample}: & 0.2 & 0.8 & 1.6,0.4,1.6,0.4\\ \mathrm{Change}\;\mathrm{Orient}\;: & 0.2 & 0.8 & 1.9,0.1,1.9,0.1\\ \mathrm{Changed}\;\mathrm{shape}\;: & 0.2 & 0.8 & 1.6,0.4,1.6,0.4\\ \mathrm{Both}\;\mathrm{changes}\;: & 0.4 & 0.6 & 1.9,0.1,1.9,0.1 \end{array}$$

Seven variables

$$\begin{array}{llll} \;\mathrm{Case} & \mathrm{Variance} & \;z_{i} & \;\left(i=1,7\right)\\ \; & s & c & \;\\ \mathrm{Refernce}\;\mathrm{sample}: & 0.2 & 0.8 & 1.3,0.6,1.3,0.6,1.3,0.6,1.3\\ \mathrm{Changed}\;\mathrm{Orient}\;: & 0.2 & 0.8 & 1.6,0.2,1.6,0.2,1.6,0.2,1.6\\ \mathrm{Changed}\;\mathrm{shape}\;: & 0.4 & 0.6 & 1.3,0.6,1.3,0.6,1.3,0.6,1.3\\ \mathrm{Both}\;\mathrm{changes}\;: & 0.4 & 0.6 & 1.6,0.2,1.6,0.2,1.6,0.2,1.6 \end{array}$$

*Detailed list of cases*

List of parameter sets used in every simulated case and resulting covariance matrices, eigenvectors and eigenvalues. The expected compositions of eigenvectors were obtained via eigenvector analyses applying R function *eigen* to random samples of size 10^6^. Note that for four and seven variables cases it was not possible to obtain a constant set of eigenvector coefficients (beyond the first eigenvector) even for such large samples. In any case, The S statistics recognized their equivalence despite differences in eigenvectors’ coefficients (see the S3 and S2 values in the second row and third rows respectively of Figure 3).

In the two variables case we had:

Reference sample:

$$y_{1}=s_{1}+\mathrm{sqrt}\left(2\right)*c$$

$$y_{1}=s_{1}+\mathrm{sqrt}\left(2\right)*c$$

where sqrt is the square root and *s*_1_ and *s*_2_ had distributions N(0, 0.2), and *c*, N(0, 0.8). The expected covariance matrix was:

$$\begin{array}{l} 1.80\;0.00\\ 0.00\;0.20 \end{array}$$

The expected eigenvectors had coefficients (columns):

$$\begin{array}{ll} \#1 & \#2\\ 1 & 0\\ 0 & 1 \end{array}$$

and the expected eigenvalues were: 1.8 and 0.2.

Compared sample with altered orientation:

$$y_{1}\;=\;s_{1\;}\;+\;\mathrm{sqrt}\left(2\right)c$$

$$x_{2}\;=\;s_{2}\;+\;\mathrm{sqrt}(0.1)c$$

where *s*_1_ and *s*_2_ had distributions N(0, 0.2), and c, N(0, 0.8). The expected covariance matrix was:

$$\begin{array}{l} 1.72\;0.35\\ 0.35\;0.28 \end{array}$$

The expected eigenvectors had coefficients (columns):

$$\begin{array}{ll} \#1 & \;\#2\\ 0.98 & \;-0.22\\ 0.22 & \;0.98 \end{array}$$

and the expected eigenvalues were: 1.8 and 0.2.

Compared sample with altered shape:

$$x_{1}\;=\;s_{1\;}\;+\;\mathrm{sqrt}\left(2\right)c$$

$$x_{1}\;=\;s_{1\;}\;+\;\mathrm{sqrt}\left(2\right)c$$

where s_1_ and s_2_ had distributions N(0, 0.5), and c, N(0, 0.5). The expected covariance matrix was:

$$\begin{array}{c} 1.50\;0.00\\ 0.00\;0.50 \end{array}$$

The expected eigenvectors had coefficients (columns):

$$\begin{array}{ll} \#1 & \#2\\ 1 & 0\\ 0 & 1 \end{array}$$

and the expected eigenvalues were: 1.5 and 0.5.

Compared sample with both orientation and shape altered:

$$x_{1}\;=\;s_{1\;}\;+\;\mathrm{sqrt}(1.9)c$$

$$x_{2}\;=\;s_{2\;}\;+\;\mathrm{sqrt}(0.1)c$$

where s_1_ and s_2_ had distributions N(0, 0.5), and c, N(0, 0.5). The expected covariance matrix was:

$$\begin{array}{c} 1.45\;0.22\\ 0.22\;0.55 \end{array}$$

The expected eigenvectors had coefficients (columns):

$$\begin{array}{ll} \#1 & \#2\\ 0.98 & -0.22\\ 0.22 & 0.98 \end{array}$$

and the expected eigenvalues were: 1.5 and 0.5.

The expected total variance in all two variable samples was = 2.

In the four variables case, we had:

Reference sample:

$$x_{1}\;=\;s_{1}+\mathrm{sqrt}\left(0.1\right)c$$

$$x_{2}\;=\;s_{2}\;+\;\mathrm{sqrt}(0.4)c$$

$$x_{3}\;=s_{3}+\mathrm{sqrt}\left(1.6\right)c$$

$$x_{4}\;=\;s_{4\;}\;+\;\mathrm{sqrt}(0.4)c$$

where *s*_1,_*s*_2,_*s*_3_ and *s*_4_ had distributions N(0, 0.2), and c, N(0, 0.8). The expected covariance matrix was:

$$\begin{array}{c} 1.48\;0.64\;1.28\;0.64\\ 0.64\;0.52\;0.64\;0.32\\ 1.28\;0.64\;1.48\;0.64\\ 0.64\;0.32\;0.64\;0.52 \end{array}$$

The expected eigenvectors had coefficients (columns):

$$\begin{array}{cccc} \#1 & \;\#2 & \;\#3 & \;\#4\\ 0.63 & \;0.03 & -0.56 & \;0.54\\ 0.32 & -0.39 & \;0.75 & \;0.43\\ 0.63 & -0.27 & \;0.00 & -0.73\\ 0.32 & \;0.88 & \;0.35 & -0.05 \end{array}$$

and the expected eigenvalues were: 3.4, 0.2, 0.2, 0.2.

Compared sample with altered orientation:

$$x_{1}\;=\;s_{1}\;+\mathrm{sqrt}(1.9)c$$

$$x_{2}\;=\;s_{2\;}\;+\;\mathrm{sqrt}(0.1)c$$

$$x_{3}\;=\;s_{3\;}\;+\;\mathrm{sqrt}(1.9)c$$

$$x_{4}\;=\;s_{4\;}\;+\;\mathrm{sqrt}(0.1)c$$

where *s*_1,_*s*_2,_*s*_3_ and *s*_4_ had distributions N(0, 0.2), and c, N(0, 0.8). The expected covariance matrix was:

$$\begin{array}{l} 1.72\;0.35\;1.52\;0.35\\ 0.35\;0.28\;0.35\;0.08\\ 1.52\;0.35\;1.72\;0.35\\ 0.35\;0.08\;0.35\;0.28 \end{array}$$

The expected eigenvectors had coefficients (columns):

$$\begin{array}{cccc} \#1 & \#2 & \#3 & \#4\\ 0.69 & -0.51 & 0.13 & 0.50\\ 0.16 & -0.01 & -0.99 & -0.04\\ 0.69 & -0.64 & 0.19 & 0.33\\ 0.16 & 0.58 & -0.01 & 0.80 \end{array}$$

and the expected eigenvalues were: 3.4, 0.2, 0.2, 0.2.

Compared sample with altered shape:

$$x_{1}\;=\;s_{1\;}\;+\;\mathrm{sqrt}(1.6)c$$

$$x_{2}\;=\;s_{2}\;+\;\mathrm{sqrt}(0.4)c$$

$$x_{3}\;=\;s_{3\;}\;+\;\mathrm{sqrt}(1.6)c$$

$$x_{4}\;=\;s_{4\;}\;+\;\mathrm{sqrt}(0.4)c$$

where *s*_1,_*s*_2,_*s*_3_ and *s*_4_ had distributions N(0, 0.4), and c, N(0, 0.6). The expected covariance matrix was:

$$\begin{array}{cccc} 1.36 & 0.48 & 0.96 & 0.48\\ 0.46 & 0.64 & 0.49 & 0.24\\ 0.96 & 0.48 & 1.36 & 0.48\\ 0.48 & 0.24 & 0.48 & 0.64 \end{array}$$

The expected eigenvectors had coefficients (columns):

$$\begin{array}{cccc} \#1 & \;\#2 & \;\#3 & \;\#4\\ 0.63 & \;0.38 & \;0.01 & -0.68\\ 0.32 & -0.01 & \;0.91 & \;0.25\\ 0.63 & -0.13 & -0.39 & \;0.66\\ 0.32 & -0.91 & -0.15 & -0.21 \end{array}$$

and the expected eigenvalues were: 2.8, 0.4, 0.4, 0.4.

Compared sample with both orientation and shape altered:

$$x_{1}\;=\;s_{1\;}\;+\;\mathrm{sqrt}(1.9)c$$

$$x_{2}\;=\;s_{2\;}\;+\;\mathrm{sqrt}(0.1)c$$

$$x_{3}\;=\;s_{3\;}\;+\;\mathrm{sqrt}(1.9)c$$

$$x_{4}\;=\;s_{4\;}\;+\;\mathrm{sqrt}(0.1)c$$

where *s*_1,_*s*_2,_*s*_3_ and *s*_4_ had distributions N(0, 0.4), and c, N(0, 0.6). The expected covariance matrix was:

$$\begin{array}{l} 1.54\;0.26\;1.14\;0.26\\ 0.26\;0.46\;0.26\;0.06\\ 1.14\;0.26\;1.54\;0.26\\ 0.26\;0.06\;0.26\;0.46 \end{array}$$

The expected eigenvectors had coefficients (columns):

$$\begin{array}{cccc} \#1 & \;\#2 & \;\#3 & \;\#4\\ 0.69 & -0.57 & \;0.09 & \;0.44\\ 0.16 & -0.27 & -0.85 & -0.42\\ 0.69 & \;0.49 & \;0.21 & -0.49\\ 0.16 & \;0.60 & -0.47 & \;0.62 \end{array}$$

and the expected eigenvalues were: 2.8, 0.4, 0.4, 0.4.

The expected total variance in all four variable samples was = 4.

In the seven variables case, we had:

Reference sample:

$$x_{1}\;=\;s_{1\;}\;+\;\mathrm{sqrt}(1.3)c$$

$$x_{2}\;=\;s_{2\;}\;+\;\mathrm{sqrt}(0.6)c$$

$$x_{3}\;=\;s_{3}\;+\;\mathrm{sqrt}(0.6)c$$

$$x_{4}\;=\;s_{4}\;+\;\mathrm{sqrt}(0.6)c$$

$$x_{5}\;=\;s_{5\;}\;+\;\mathrm{sqrt}(1.3)c$$

$$x_{6}\;=\;s_{6\;}\;+\;\mathrm{sqrt}(0.6)c$$

$$x_{7}\;=\;s_{7\;}\;+\;\mathrm{sqrt}(1.3)c$$

where *s*_1,_*s*_2,_*s*_3,_*s*_4,_*s*_5,_*s*_6,_ and *s*_7_ had distributions N(0, 0.2), and c, N(0, 0.8). The expected covariance matrix was:

$$\begin{array}{l} 1.24\;0.71\;1.04\;0.71\;1.04\;0.71\;1.04\\ 0.71\;0.68\;0.71\;0.48\;0.71\;0.48\;0.71\\ 1.04\;0.71\;1.24\;0.71\;1.04\;0.71\;1.04\\ 0.71\;0.48\;0.71\;0.68\;0.71\;0.48\;0.71\\ 1.04\;0.71\;1.04\;0.71\;1.24\;0.71\;1.04\\ 0.71\;0.48\;0.71\;0.48\;0.71\;0.68\;0.71\\ 1.04\;0.71\;1.04\;0.71\;1.04\;0.71\;1.24 \end{array}$$

The expected eigenvectors had coefficients (columns):

$$\begin{array}{ccccccc} \;\#1 & \;\#2 & \;\#3 & \;\#4 & \;\#5 & \;\#6 & \;\#7\\ -0.43 & \;0.05 & -0.08 & -0.68 & \;0.08 & \;0.00 & \;0.58\\ -0.29 & -0.40 & \;0.22 & \;0.07 & \;0.80 & \;0.26 & \;0.04\\ -0.43 & \;0.31 & \;0.50 & -0.13 & -0.24 & \;0.41 & -0.47\\ -0.29 & \;0.45 & -0.73 & \;0.16 & \;0.23 & \;0.27 & -0.14\\ -0.43 & -0.32 & -0.20 & -0.16 & -0.03 & -0.62 & -0.51\\ -0.29 & \;0.51 & \;0.33 & \;0.41 & \;0.17 & -0.52 & \;0.29\\ -0.43 & -0.42 & -0.10 & \;0.54 & -0.47 & \;0.20 & \;0.28 \end{array}$$

and the expected eigenvalues were: 5.8, 0.2, 0.2, 0.2, 0.2, 0.2, 0.2.

Compared sample with altered orientation:

$$x_{1}\;=\;s_{1\;}\;+\;\mathrm{sqrt}(1.6)c$$

$$x_{2}\;=\;s_{2\;}\;+\;\mathrm{sqrt}(0.2)c$$

$$x_{3}\;=\;s_{3\;}\;+\;\mathrm{sqrt}(1.6)c$$

$$x_{4}\;=\;s_{4\;}\;+\;\mathrm{sqrt}(0.2)c$$

$$x_{5}\;=\;s_{5\;}\;+\;\mathrm{sqrt}(1.6)c$$

$$x_{6}\;=\;s_{6\;}\;+\;\mathrm{sqrt}(0.2)c$$

$$x_{7}\;=\;s_{7\;}\;+\;\mathrm{sqrt}(1.6)c$$

where *s*_1,_*s*_2,_*s*_3,_*s*_4,_*s*_5,_*s*_6,_ and *s*_7_ had distributions N(0, 0.2), and c, N(0, 0.8). The expected covariance matrix was:

$$\begin{array}{l} 1.48\;0.45\;1.28\;0.45\;1.28\;0.45\;1.28\\ 0.45\;0.36\;0.45\;0.16\;0.45\;0.16\;0.45\\ 1.28\;0.45\;1.48\;0.45\;1.28\;0.45\;1.28\\ 0.45\;0.16\;0.45\;0.36\;0.45\;0.16\;0.45\\ 1.28\;0.45\;1.28\;0.45\;1.48\;0.45\;1.28\\ 0.45\;0.16\;0.45\;0.16\;0.45\;0.36\;0.45\\ 1.28\;0.45\;1.28\;0.45\;1.28\;0.45\;1.48 \end{array}$$

The expected eigenvectors had coefficients (columns):

$$\begin{array}{lllllll} \#1 & \;\#2 & \;\#3 & \;\#4 & \;\#5 & \;\#6 & \;\#7\\ -0.5 & \;0.47 & \;0.52 & -0.1 & -0.1 & \;0.07 & \;0.50\\ -0.2 & \;0.03 & \;0.38 & \;0.3 & -0.5 & -0.24 & -0.61\\ -0.5 & \;0.01 & -0.70 & \;0.2 & -0.5 & \;0.06 & \;0.17\\ -0.2 & \;0.25 & -0.04 & -0.1 & \;0.1 & \;0.81 & -0.47\\ -0.5 & -0.06 & -0.10 & -0.6 & \;0.3 & -0.42 & -0.33\\ -0.2 & -0.81 & \;0.28 & -0.2 & -0.2 & \;0.32 & \;0.15\\ -0.5 & -0.23 & \;0.07 & \;0.6 & \;0.6 & -0.02 & -0.01 \end{array}$$

and the expected eigenvalues were: 5.8, 0.2, 0.2, 0.2, 0.2, 0.2, 0.2.

Compared sample with altered shape:

$$x_{1}\;=\;s_{1\;}\;+\;\mathrm{sqrt}(1.3)c$$

$$x_{2}\;=\;s_{2\;}\;+\;\mathrm{sqrt}(0.6)c$$

$$x_{3}\;=\;s_{3\;}\;+\;\mathrm{sqrt}(0.6)c$$

$$x_{4}\;=\;s_{4\;}\;+\;\mathrm{sqrt}(0.6)c$$

$$x_{5}\;=\;s_{5\;}\;+\;\mathrm{sqrt}(1.3)c$$

$$x_{6}\;=\;s_{6\;}\;+\;\mathrm{sqrt}(0.6)c$$

$$x_{7}\;=\;s_{7\;}\;+\;\mathrm{sqrt}(1.3)c$$

where s_1,_ s_2,_ s_3,_ s_4,_ s_5,_ s_6,_ and s_7_ had distributions N(0, 0.4), and c, N(0, 0.6). The expected covariance matrix was:

$$\begin{array}{l} 1.18\;0.53\;0.78\;0.53\;0.78\;0.53\;0.78\\ 0.53\;0.76\;0.53\;0.36\;0.53\;0.36\;0.53\\ 0.78\;0.53\;1.18\;0.53\;0.78\;0.53\;0.78\\ 0.53\;0.36\;0.53\;0.76\;0.53\;0.36\;0.53\\ 0.78\;0.53\;0.78\;0.53\;1.18\;0.53\;0.78\\ 0.53\;0.36\;0.53\;0.36\;0.53\;0.76\;0.53\\ 0.78\;0.53\;0.78\;0.53\;0.78\;0.53\;1.18 \end{array}$$

The expected eigenvectors had coefficients (columns):

$$\begin{array}{lllllll} \;\#1 & \;\#2 & \;\#3 & \;\#4 & \;\#5 & \;\#6 & \;\#7\\ -0.43 & -0.43 & -0.39 & -0.52 & \;0.02 & \;0.46 & \;0.04\\ -0.29 & -0.41 & \;0.46 & -0.20 & \;0.47 & -0.49 & -0.20\\ -0.43 & \;0.26 & -0.64 & \;0.13 & \;0.04 & -0.56 & -0.08\\ -0.29 & \;0.04 & \;0.31 & -0.25 & -0.65 & -0.29 & \;0.50\\ -0.43 & -0.34 & \;0.09 & \;0.78 & -0.02 & \;0.22 & \;0.20\\ -0.29 & \;0.14 & \;0.20 & \;0.02 & -0.44 & \;0.14 & -0.80\\ -0.43 & \;0.67 & \;0.28 & -0.10 & \;0.40 & \;0.31 & \;0.18 \end{array}$$

and the expected eigenvalues were: 4.6, 0.4, 0.4, 0.4, 0.4, 0.4, 0.4.

Compared sample with both orientation and shape altered:

$$x_{1}\;=\;s_{1\;}\;+\;\mathrm{sqrt}(1.6)c$$

$$x_{2}\;=\;s_{2\;}\;+\;\mathrm{sqrt}(0.2)c$$

$$x_{3}\;=\;s_{3\;}\;+\;\mathrm{sqrt}(1.6)c$$

$$x_{4}\;=\;s_{4\;}\;+\;\mathrm{sqrt}(0.2)c$$

$$x_{5}\;=\;s_{5\;}\;+\;\mathrm{sqrt}(1.6)c$$

$$x_{6}\;=\;s_{6\;}\;+\;\mathrm{sqrt}(0.2)c$$

$$x_{7}\;=\;s_{7}\;+\;\mathrm{sqrt}(1.6)c$$

where s_1,_ s_2,_ s_3,_ s_4,_ s_5,_ s_6,_ and s_7_ had distributions N(0, 0.4), and c, N(0, 0.6). The expected covariance matrix was:

$$\begin{array}{l} 1.36\;0.34\;0.96\;0.34\;0.96\;0.34\;0.96\\ 0.34\;0.52\;0.34\;0.12\;0.34\;0.12\;0.34\\ 0.96\;0.34\;1.36\;0.34\;0.96\;0.34\;0.96\\ 0.34\;0.12\;0.34\;0.52\;0.34\;0.12\;0.34\\ 0.96\;0.34\;0.96\;0.34\;1.36\;0.34\;0.96\\ 0.34\;0.12\;0.34\;0.12\;0.34\;0.52\;0.34\\ 0.96\;0.34\;0.96\;0.34\;0.96\;0.34\;1.36 \end{array}$$

The expected eigenvectors had coefficients (columns):

$$\begin{array}{l} \begin{array}{lllllll} \;\#1 & \;\#2 & \;\#3 & \;\#4 & \;\#5 & \;\#6 & \;\#7\\ -0.48 & -0.11 & \;0.36 & \;0.27 & \;0.59 & -0.25 & -0.38\\ -0.17 & \;0.20 & -0.00 & \;0.02 & \;0.46 & \;0.77 & \;0.37\\ -0.48 & \;0.37 & -0.57 & -0.50 & \;0.07 & -0.13 & -0.21\\ -0.17 & \;0.44 & -0.07 & \;0.60 & -0.42 & \;0.27 & -0.39\\ -0.48 & -0.54 & \;0.21 & -0.28 & -0.43 & \;0.37 & -0.15\\ -0.17 & \;0.55 & \;0.67 & -0.28 & -0.22 & -0.16 & \;0.26\\ -0.48 & -0.14 & -0.22 & \;0.39 & -0.16 & -0.30 & \;0.66 \end{array} \end{array}$$

and the expected eigenvalues were: 4.6, 0.4, 0.4, 0.4, 0.4, 0.4, 0.4.

The expected total variance in all seven variable samples was = 7.

## Competing interests

The author declares that he has no competing interests.
